# Noise utilization as an approach for reducing energy consumption in street lighting

**DOI:** 10.1371/journal.pone.0219373

**Published:** 2019-07-11

**Authors:** Yasser A. Farghaly, Fahd Abdel Aziz Hemeida, Sahar Salah

**Affiliations:** Department of Architectural Engineering & Environmental Design, College of Engineering and Technology, Arab Academy and Science and Technology and Maritime Transport, Alexandria, Egypt; University of Maryland Baltimore County, UNITED STATES

## Abstract

Noise is considered as one of the challenging problems in big cities. However, this noise could be utilized in producing energy especially in dense urban areas. Sound as a form of mechanical energy, it can be converted to electric energy through many approaches including heating, by using the diaphragm and through using piezoelectric materials. This research aims at utilizing noise through using piezoelectric materials as an approach of conversion to produce green sustainable electric energy that can be used to decrease the energy consumption from non-renewable sources and utilizing this energy in street lighting. The study was carried in three bus stations in Alexandria by having measurements during weekdays and weekends in order to study the noise produced in the selected stations and the amount of electric energy that could be produced and utilized in street lighting. The noise level index **L**_**DEN**_ was calculated for each of the three selected locations. The equivalent noise level values were always exceeding the limits through the day, evening and night. At daytime they ranged between 75–85 dB which is higher that the permissible limit by 10–20 dB, at evening they ranged from 80–85 dB which is also higher than the permissible limit with 20–25 dB and at the night they ranged from 75–80 dB which is higher by 20–25 dB than the permissible limit. The research concluded that utilizing noise using the piezoelectric material is successful. The electric energy produced from an area of 1.45 m2 containing 690 piezoelectric QB220-503YB transducers at each of the selected stations was about 0.024 watt hr. This amount of electric energy is too small to be used in an application. So the application area should be maximized to hundreds of square meters to produce beneficial electric energy that can be used in lighting 1 LED street lamp or it can be stored and used when needed in applications that use greater amount of electric energy and this would help in reducing the energy consumed.

## Introduction

Environmental pollution is a major problem facing all countries around the world. Rapid growth on the industrial and urban side has concluded in vast quantities of potentially harmful waste products being related to the environment. The massive increase in the number of inhabitants and vehicles has led to major concern about "*Noise Pollution*" where it has become a major problem facing societies [1]. Noise pollution is impacting on the communities’ developments as it has a direct and indirect effect on human activities like education, production, economic processes and social aspects. Noise effects have different impacts on human’s mental and physical health and disturbance of daily activities and that effects can cause temporary or permanent hearing loss, ranging from slight impairment to nearly total deafness [2].

Traffic noise is considered as the major source of noise pollution in cities [3]. All these means of transportation such as airplanes, buses, trains, heavy trucks and motor-cycles increase the noise pollution [4]. Noise is a kind of energy but with sound pressure levels higher than the levels that are audible by human ear. According to the third law of thermodynamics, mechanical energy can be converted to electrical energy, thus sound as a form of mechanical energy can be converted to electric energy. There are three approached to convert sonic energy to electric energy; by heating, by using the diaphragm and through using piezoelectric materials. [5] [6]. This research will focus on utilizing noise through using piezoelectric materials as an approach of conversion to produce green sustainable electric energy that can be used to decrease the energy consumption produced from non-renewable sources.

## Literature review

Referencing to the law of conservation of energy which states that "Energy cannot be created nor be destroyed", sound waves as a form of mechanical energy can be converted to other forms such as heat energy or electric energy through choosing the suitable approach of conversion [7]. Noise can be described as unwanted sound waves [8] are originated from human activities, especially urbanization and the development of transport systems. Though the urban population is much more affected by such pollution, small towns along side roads or industrial areas are also affected by this pollution [9]. The source of most outdoor noise worldwide is transportation systems including roads, flyways, and rail traffic which are called traffic noise where people living in urban city complained about the noise from buses, trains, heavy trucks, motor-cycles, airplanes and pneumatic drills. These sources produced noise ranged from 85 to 120 dB [10].

The World Health Organization (WHO) in 2011, points out that noise pollution ranked second among a series of environmental stressors for public health impact [11]. In addition to that the WHO has set a pyramid that indicated the severity of noise pollution effect on human beings health. In the last decades, it has been observed that the noise pollution levels in Egyptian cities are rapidly increasing. The noise pollution issue in Egypt ranks in the second place among the environmental pollution issues as stated by the Egyptian Environmental Affairs Agency in its complaint survey in 2006. Cairo government the capital of Egypt is ranked in the third place of the worst ten cities for noise pollution [12].

In 1992, Egypt was nominated one of a major environmental clean-up policy as it charged the Ministry of State for Environmental Affair to institute the Egyptian Environmental number 4 law in 1994 and the ministry was responsible for its execution regulation. The law 4/1994 identified the maximum permissible noise limits for different land uses as shown in Table 1 [13].

**Table 1:** Egyptian noise standards and policy on the maximum permissible limit for noise intensity Leq (dB) in different land use areas according to the Egyptian Environmental Law No. 4/1994 [13].

Type of area	Permissible limit for noise intensity (dB)		
	Day (7 am–6 pm)	Evening (6–10 pm)	Night (10 pm–7 am)
Rural residential areas, hospitals and gardens	45	40	35
Residential suburbs with low traffic	50	45	40
City residential areas	55	50	45
Residential areas with commercial establishments	60	55	50
Commercial, administrative and downtown areas.	65	60	55
Industrial areas (heavy industry)	70	65	60

Noise levels measurement in most of Egyptian streets and squares range from 75 dB to 85 dB, which is higher than the maximum permissible noise levels that, was stipulated in law 4/1994 on the environment and its executive regulations. Population growth, associated activities and lack of sound urban planning are the major causes of high noise levels in most of cities and capitals. [13] [14].

Egypt has a big source of wasted energy in the form of noise pollution as an unwanted sound. This wasted energy could be converted to a beneficial source of energy through using the suitable approach with the appropriate technology. As mentioned earlier in this research that according to the third law of thermodynamics, mechanical energy can be converted to electrical energy, thus sound as a form of mechanical energy can be converted to electric energy. There are three approaches to convert sound to electric energy; by heating, by using the diaphragm and through using piezoelectric materials. [6] [5].

Piezoelectricity is the electricity resulting from pressure. This happens by using crystals to convert mechanical energy to electric energy or vice versa. Its scientific definition is the appearance of an electrical potential (a voltage) across the sides of a crystal when you subject it to mechanical stress [15]. When the crystal is deformed by the application of an external stress, electric charges appear on the crystal surfaces; and when the direction of the strain reverses, the polarity of the electric charge is reversed. This is called the direct piezoelectric effect. Inversely, when a piezoelectric crystal is placed in an electric field, or when charges are applied by external means to its faces, the crystal exhibits strain, i.e. the dimensions of the crystal changes. When the direction of the applied electric field is reversed, the direction of the resulting strain is reversed and this is called the inverse piezoelectric effect. [5]. There are different types of piezoelectric materials, some are naturally found such as Quartz, Rochelle Salt, Topaz, Sucrose, Enamel/ Dentin, Bones and Tourmaline and some are man-made synthetic ones such as Lead Zirconate Titanate (PZT), Barium Titanate (BaTiO3), Gallium Orthophosphate (GaPO_4_), Zinc oxide (ZnO), Polyvinylidene Fluoride (PVDF) and in Table 2 there is an investigation about the advantages, disadvantages and power harvesting capabilities for some piezoelectric materials transducers’ [16] [17].

**Table 2:** Summary of some piezoelectric materials transducers’ investigation about their advantages and disadvantages and their power harvesting capabilities [18] [19].

Type Of Material	Advantages/Disadvantages	Power Harvesting Capabilities
**Monolithic PZT**	Most common type of device, not flexible, susceptible to fatigue crack, growth during cyclic loading.	N/A
**PVDF film coated with PEDOT/PSS electrodes**	Resistance to fatigue crack damage to electrodes.	N/A
**Piezofiber composite**	Increased flexibility	120 mW from 34 × 11mm plate of 5.85 mm thickness
**Piezofiber composite**	Increased flexibility	7.5 mW from 130 × 13 mm patch of 0.38 mm thickness
**MFC composite quick pack IDE quick pack**	MFC: flexibility, MFC and quick pack IDE: low-capacitance devices, quick pack: energy harvesting capability	Quick pack proved to harvest the most energy
**Monolithic PZT, quick pack, MFC**	MFC: flexibility quick pack and monolithic PZT: energy harvesting capability	PZT proved to be most efficient (6.8% for random vibration excitation)

Piezoelectric material experiences strain when an electrical field is applied to it and develops an internal electric field. The relationship between the electrical and elastic properties of the material can be expressed by the following equations: D = d T + ɛ^T^ E and S = s^E^ T + d E Where (*D*) is electric flux density or dielectric displacement, (*T*) is the mechanical stress, (*E*) is the electric field strength, (*S*) is the mechanical strain, (*d*) is the piezoelectric charge coefficient, (*ɛ*^*T*^) is the dielectric permittivity for constant T and (*s*^*E*^) is the elastic coefficient for constant E. These small-signal coefficients can be found in the material data table for each material [20].

Lead Zirconate Titanate (PZT) and Lead Titanate are from the most commonly used piezoelectric materials that are used nowadays as they have large Curie temperature and high efficiency and they are less expensive and easier to machine in wide range of shapes and sizes than single crystals like Quartz.

Piezoelectric materials that are used in architectural applications belong to the group of adaptive material or so called "Smart Materials" which possess both transducers and actuators characteristics. They are a thin piezo-ceramic film which is covered with electrically conducting material to make electrical contact and are subsequently embedded in an elastic polymer composite [21]. The transducers are simply glued to the corresponding building structure and façades or integrated in to the structure material during manufacture to detect vibrations in the component itself. They detect any change in the surrounding environmental conditions such as impact pressure or bending loads and react to them. Even under high dynamic stress or pressure the construction guarantees high damage tolerance, reliability and a life time of more than 10^9^ cycles. They have low susceptibility to defect because they don’t contain any moving parts [21].

**Fig 1:**
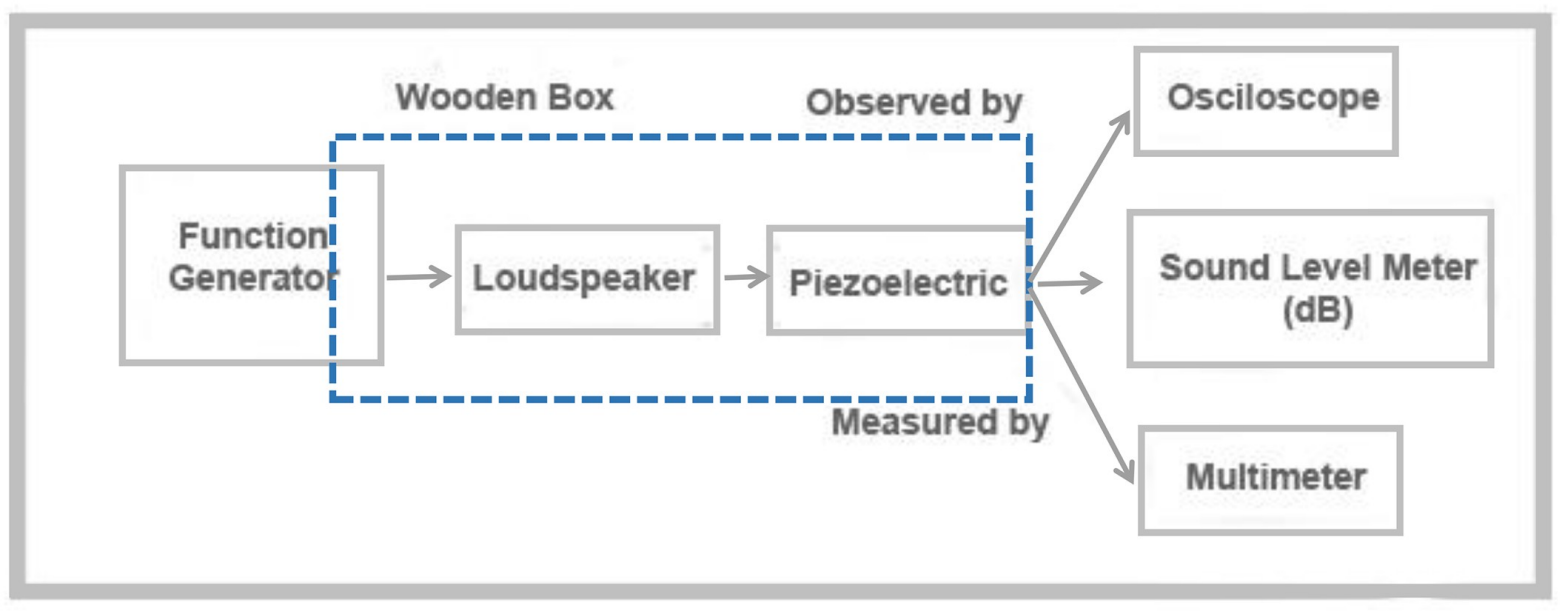
The experimental setup for characteristics of the piezoelectric strip harvester on sound wave [22].

As shown in Table 2, after the various studies about the properties of piezoelectric materials transducers’ PZT transducers were the best to use in harvesting vibrations produced from different kinds of mechanical energies and therefore sound energy as if formed a kind of mechanical energy. Liew Hui Fanga study’s titled with *Characterization of Different Dimension Piezoelectric Transducer for Sound Wave Energy Harvesting* made an investigation experiment as shown in Fig 1 using a various type of piezoelectric transducers based on energy harvesting system to convert environmental acoustic vibration (noise/ sound) into electric energy. The varieties of piezoelectric transducer structures are targeted to resonate at the ambient frequency which is below 1 kHz. The experimented sound level of the piezoelectric is at the range of 35–100 dB. It is comparable with ambience environmental human sound of level 50 dB to 100 dB.

**Fig 2:**
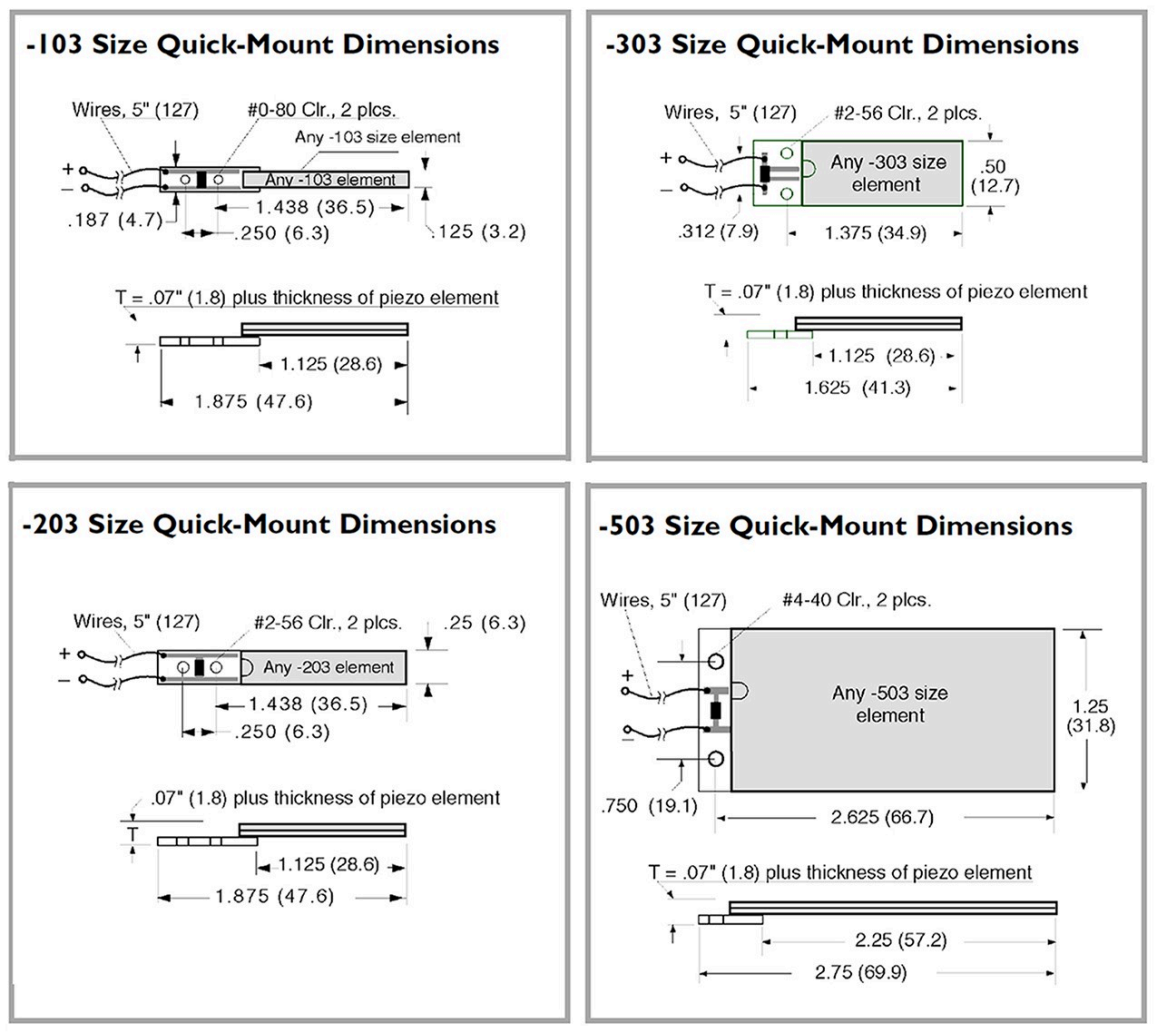
The variety of some of the piezoelectric transducer in custom configurations and sizes designs [22] [23].

**Fig 3:**
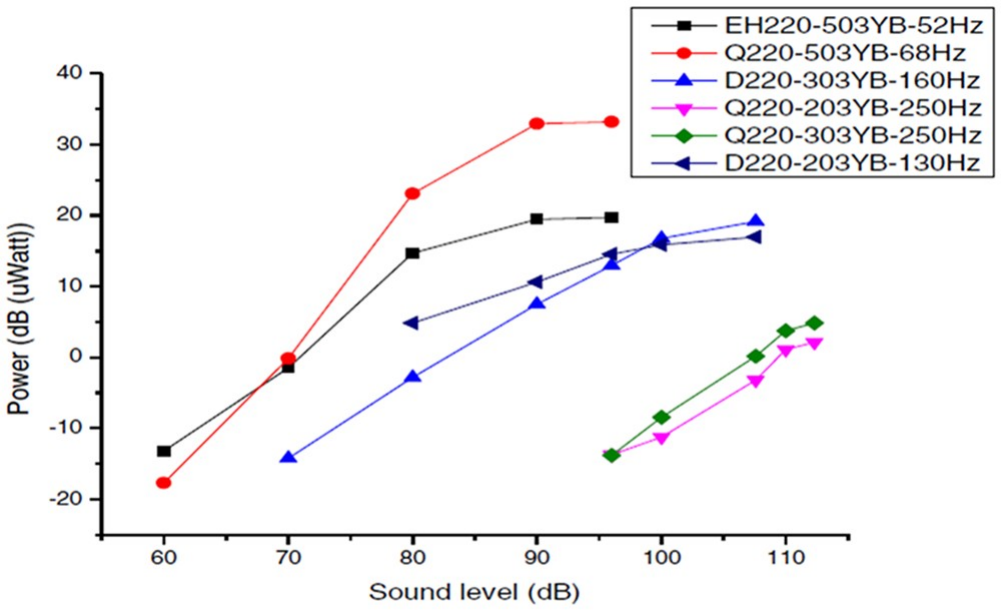
The graph of power versus sound level for variety type piezoelectric transducers [22].

The piezoelectric transducers used through the investigation were (Q220-203YB, Q220-303YB, Q220-503YB, D220-303YB, D220-503YB and EH220-503YB) and some of their profile configurations and sizes are shown in Fig 2 [22] [23]. Through the experiment these transducers were acts as a transducer, which converts the vibration from the sound waves to AC electricity. The results of variety piezoelectric transducer to harvest and convert the sound wave energy are shown in Table 3 and the plotted graph is shown in Fig 3 [22].

**Table 3:** The value of power versus sound level for variety type’s piezoelectric transducer used through the experiment [22].

Sound Level (dB)	10 log (Power) dBμW					
	52 Hz	68 Hz	160 Hz	250 Hz	250 Hz	130 Hz
	EH220- 503YB	Q220- 503YB	D220- 303YB	Q220- 203YB	Q220- 303YB	D220- 203YB-
35	*NIL*	*NIL*	*NIL*	*NIL*	*NIL*	*NIL*
40	*NIL*	*NIL*	*NIL*	*NIL*	*NIL*	*NIL*
50	*NIL*	*NIL*	*NIL*	*NIL*	*NIL*	*NIL*
60	-13.188	-17.7	*NIL*	*NIL*	*NIL*	*NIL*
70	-1.463	-0.1265	-14.202	*NIL*	*NIL*	*NIL*
80	14.687	23.05	-2.816	*NIL*	*NIL*	4.857
90	19.44	32.902	7.494	*NIL*	*NIL*	10.624
96	19.69	33.169	12.937	-13.737	-13.809	14.53
100	19.69	33.169	16.772	-11.249	-8.444	15.851
107.6	19.69	33.169	19.146	-3.188	0.16	16.96
110	19.69	33.169	19.146	1.106	3.765	16.96
112.5	19.69	33.169	19.146	2.143	4.8458	16.96

The QB 220-A4-503YB type has shown a better performance of output power and sound level value. The large size of the piezoelectric transducer is able to convert a higher mechanical energy from sound wave into electrical energy. An energy harvester of piezoelectric transducer accomplished constant stage with the maximum amplitude at sound level of 96dB. It accomplished maximum power response performance at the 96 dB with 33.133 dB (μW) as shown in Table 4. Thus, the results show that the customs size, structure and the material properties of piezoelectric will affect the efficiencies of the transducers to harvest energy [22].

**Table 4:** The detailed values of current, voltage and power for piezoelectric transducer QB220-503YB that is found in the data sheet of the material [22] modified by researcher.

Sound Level (dB)	Current Isc (mA)	Voltage Vop (Vrms)	Power Watt (μW)	10 log (Power) dBμ
**35**	0	0	0	*NIL*
**40**	0	0	0	*NIL*
**50**	0	0.0023	0	*NIL*
**60**	0.001	0.017	0.017	-17.70
**70**	0.0103	0.0943	0.9713	-0.1265
**80**	0.1753	1.213	212.6	23.28
**90**	0.5237	3.7054	1940.52	32.0879
**96**	0.5283	3.894	2057.2	33.153

PZT transducer has many features that make it capable of converting sound energy to electric energy; it has huge efficient piezoelectric material that can convert up to 80% of mechanical energy to electricity, it has high piezoelectric voltage and high voltage conversion, it can generate power that can be stored in a battery as DC power. The generated electrical power can instantly be used to power microelectronic devices like Bluetooth, GPS modules, micro-controllers and low power units. As the material is quite robust it can be embedded in a variety of structures, on walls, under floors, roads, sidewalks and street furniture. [18] [19].

Noise as a part of urban environment and our everyday life and it is one of the most prevalent pollution forms in cities, it is also could be utilized as an important source of energy not valued yet [24]. In 2013, one of the honorable mentions winners in eVolo 2013 Skyscraper Competition was given to *Julien Bourgeois*, *Olivier Colliez*, *Savinien de Pizzol*, *Cédric Dounval*, *Romain Grouselle* from France for designing a soundscraper that takes advantage of city noise pollution by capturing airborne sound and converting it into usable electric energy. The Sound-scraper would be constructed next to main transport infrastructures, motorways and railroads junctions mostly inside city centers where noise pollution is at its maximum. The skyscraper’s skin consists of electro active lashes supported by a light metallic structure that form the exterior of the building [24]. The lashes are covered with sound sensors P.F.I.G (Parametric Frequency Increased Generators) which are made of piezoelectric material. These specialized PFIG energy harvester convert sounds vibrations caused by surrounding noises to capture mechanical kinetic energy, after which an array of piezoelectric transducer "cells" employing a novel actuation method are used to convert the mechanical energy into electricity. The electrical current is then transferred to a main storage to be stored or distributed to the grid for regular electric use through the city [24] [25].

Also The Urban Transducer Skyscraper was a design competition entry in Evolo Skyscraper Competition in 2009 as a mixed-use high rise. It was desgined by *Ryan Browne*, *Nathanael Dunn*, *Daniel Nelson and Benjamin Scholten* from United States of America. [26]. The Urban Transducer utilizes city noise pollution in Chicago by capturing sound and converting it into usable electric energy, and using that energy to help power the skyscraper. In order to capture the maximum amount of energy from noise, acoustic panels that perceive present frequencies and tune bands to match the extant wavelengths, creating resonance through which energy is harvested from the noise [27].

The panels also save the most recurrent frequencies at their location, allowing preemptive adjustment to maximize efficiency of energy conversion. Each panel has a piezoelectric transducer that transforms the sound vibrations into an electrical current that is stored in a unit at the end of each band and then transferred to a building storage compartment that regulates the tower’s electrical needs [27].

**Fig 4:**
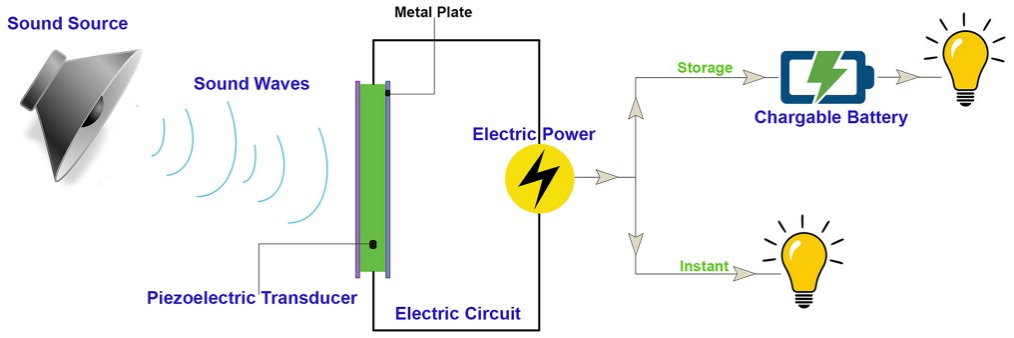
A diagram of sonic energy conversion process. (Researcher, 2018).

The common elements of sound energy harvesting system include an input mechanical power spectrum (noise/sound), a piezoelectric transducer, an electric circuit and charges’ store as presented in Fig 4 [28].

Through the previous literature review sonic energy utilization as a new and clean source of energy through the smart material technologies of piezoelectric material could help as a sustainable solution for reducing the energy consumption.

## Methodology

**Fig 5:**
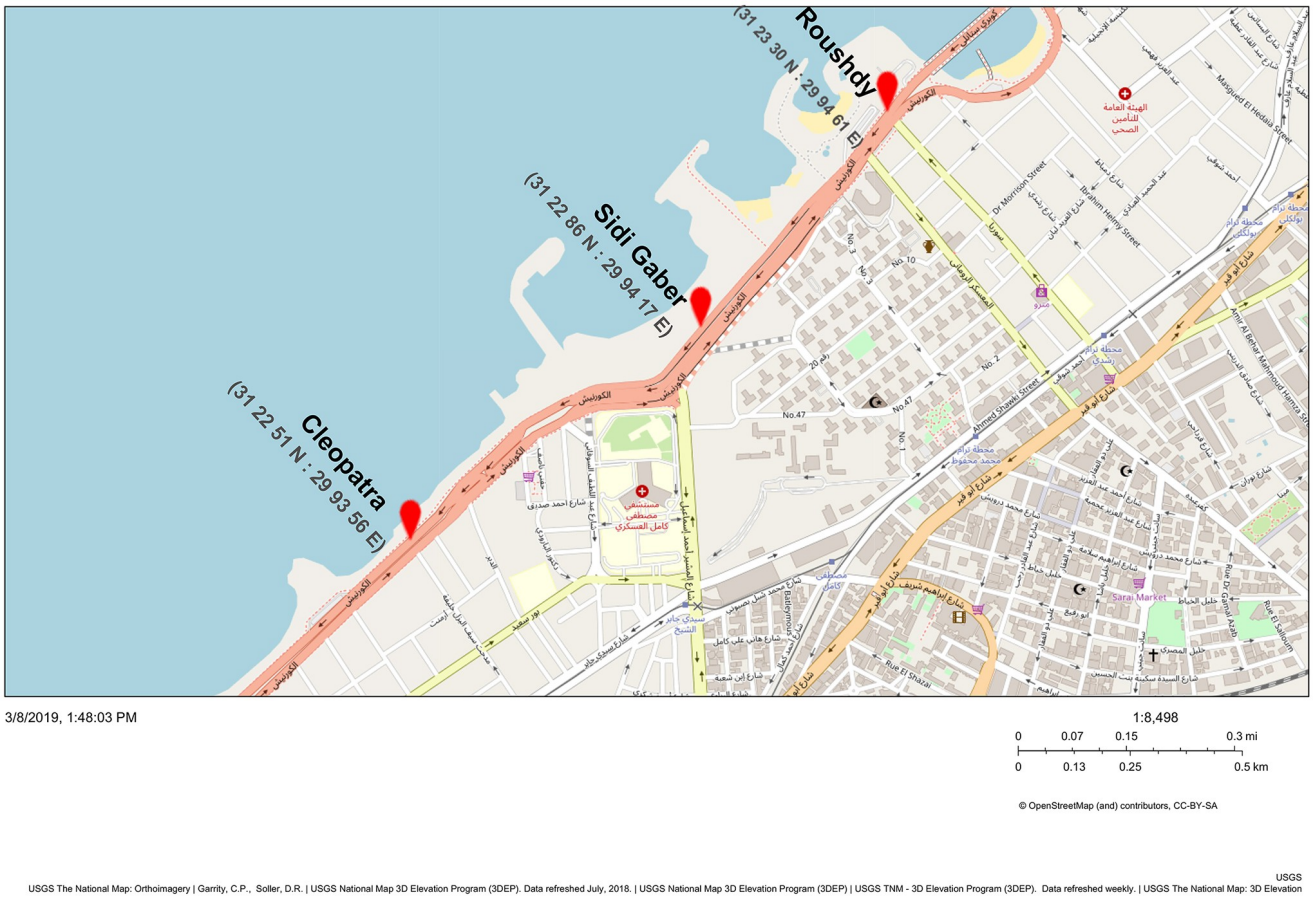
The three selected stations along Elgiesh Roadway in Alexandria City, Egypt. (The National Map Advanced Viewer, 2019).

A case study was carried in Alexandria that is considered to be the second capital of Egypt. The total area of Alexandria is about 2818.77 km^2^ with total population of 5,163,441 and population density of 1831 per square kilometer according to the Central Agency for Public Mobilization and Statistics [29]. Due to this high population density, the city is characterized by high traffic rate which has a great impact on noise level. The study was held in Elgeish road as one of the main roadways in Alexandria. It links the city from its east side starting from El Montaza station to its west at El Anfoshy station. This roadway always has high traffic stream and high noise level. For evaluation of noise levels in Elgeish road, three sample stations are selected along the roadway length to be monitored in this research. The stations are: Roushdy (ROS), Sidi Gaber (SGS) and Cleopatra (CLS) stations along the northern side of Elgeish roadway according to bus stops as presented in Fig 5. This side of the street is selected for the study as most of citizens’ activities such as running and cycling are done on this side of the street and it has wider sidewalk and most of vendors, coffee shops and change in the land use through the last 5 years happened at this side. No specific permissions were required for these locations as they are open public street stations in the city’s street and the field studies did not involve endangered or protected species.

The noise measurements are carried out at the three selected stations on working days (Monday to Thursday) as they are the most crowded days, in addition to taking noise level readings on a weekend (Friday) at Cleopatra Station (CLS) as a sample to compare the resulting readings between working days and weekends. The measurements timing is selected according to the International Organization for Standardization (ISO) [30] [31] [32] and the Egyptian Environmental Law No. 4/1994 where each time of the day has its permissible noise level as shown in Table 5. The permissible limit (PL) for noise pressure at day is 65 dB, at evening is 60 dB while at night is 55 dB at the commercial, administrative and downtown areas and streets.

**Table 5:** The measurements timing that are selected for the study.

Time	(PL) for noise pressure (dB)	ISO & Egyptian law Classifications	Selected Measurement Time
Day	65	7 am- 6 pm	D1: 7–9 am, D2: 11 am-1 pm & D3: 3–5 pm
Evening	60	6 pm– 10 pm	E1: 7–9 pm
Night	55	10 pm- 7 am	N1: 11 pm- 1 am & N2: 3 - 5am

**Fig 6:**
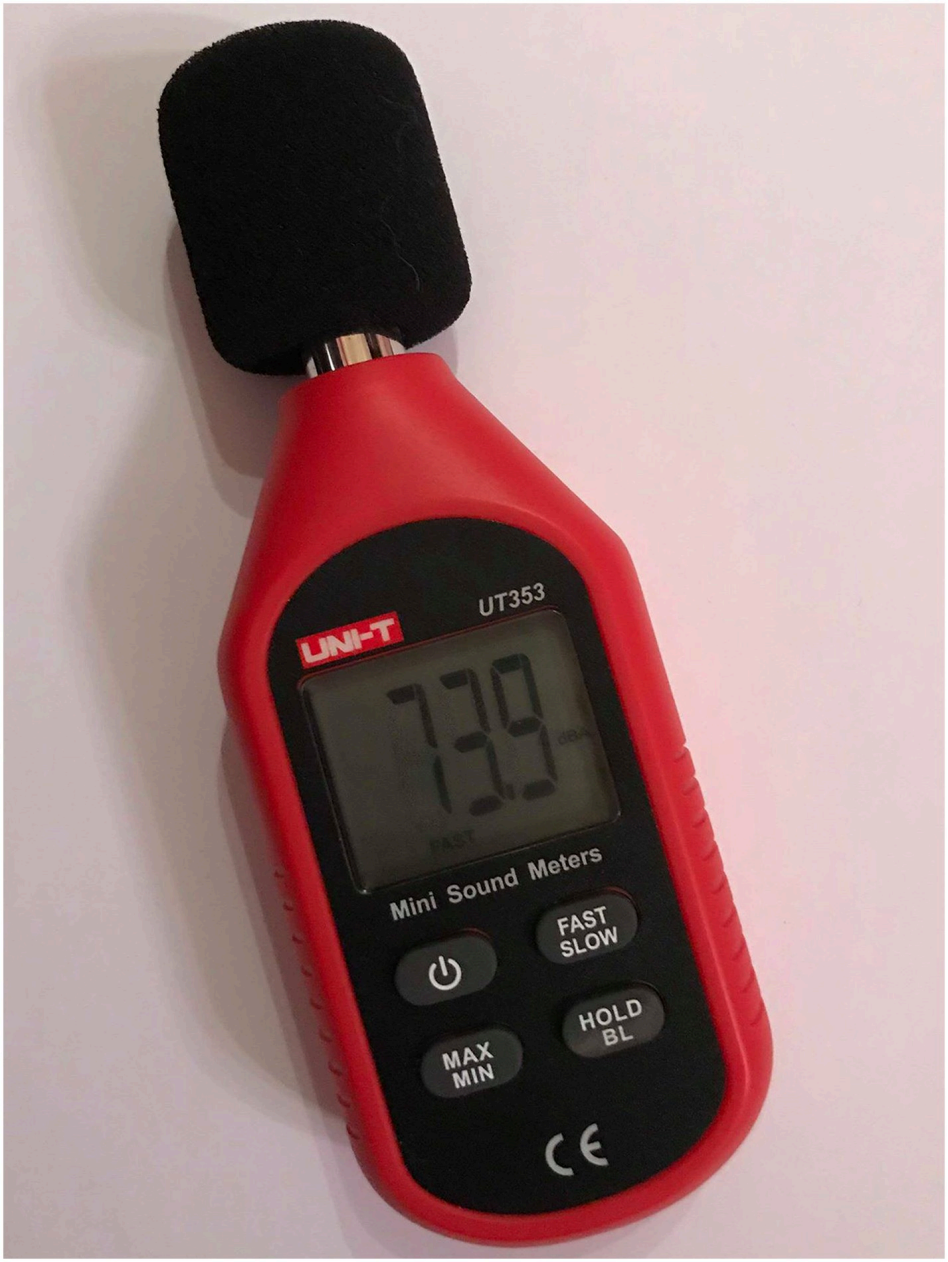
The UT 353 digital sound meter device. (Researcher, 2018).

The selected measuring interval is 2 hours in day, evening and night. Daytime is from 7am to 6pm where (D1) from 7–9 am, (D2) from 11 am-1 pm and (D3) from 3–5 pm. In evening time (E1) is from 7pm to 9 pm and at nighttime where (N1) from 11 pm- 1 am and (N2) from 3 - 5am. The readings are taken every 15 minutes using UT 353 digital sound meter device shown in Fig 6 giving 8 readings at each interval for each time of the day at each selected station. In all cases, the measuring device was placed at height 100 cm from sidewalk floor.

The mean equivalent sound pressure level (L_eq_), the maximum sound pressure level (L_max_) and the minimum sound pressure level (L_min_) in (dB) is calculated for each time of the day at the selected stations.

The mean equivalent sound pressure level (L_eq_) is calculated by using Microsoft Excel software for this equation: Leq = 10 log ((1/n) ∑ i = 1 10 (Leq/ 10)) (dB) where (n) is the number of readings measurements in the selected time interval for each station [33], while the maximum and minimum sound pressure levels are measured through the noise level measuring device. Then LDEN is calculated for the mean, maximum and minimum using the following equation for each station (1): ${\text{L}}_{\text{DEN}}=10\;\text{log}\frac{1}{24}{\left(11*10^{L_{D}}+4*10^{L_{E+5}}+9*10^{L_{N+10}}\right)}^{\frac{1}{10}}$ (dB) [34].

**Fig 7:**
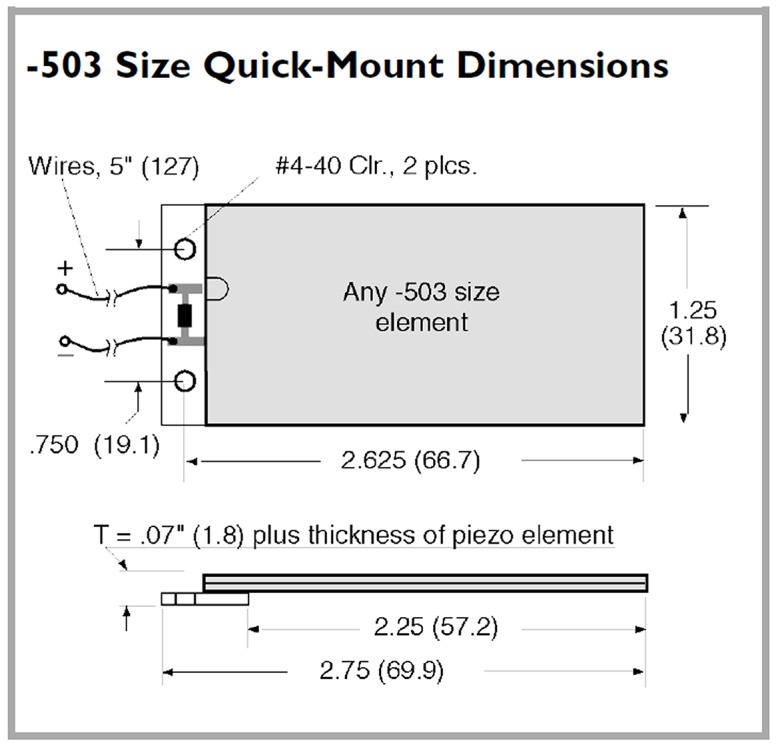
The configuration and the dimensions of piezoelectric transducer QB220-503YB [23].

To calculate the electric energy possible to be produced at each of the case study selected station, a suitable piezoelectric material must be chosen to convert the mean equivalent L_DEN_ produced at each station through the day. The QB220-503YB type is the piezoelectric transducer is chosen (Fig 7) for this case as their surfaces are large with dimensions (31.25 mm * 2.63mm and with thickness 1.8mm) and its physical characteristics are presented in Table 6 which is able to harvest more noise, it has shown a great performance of producing output power through experiments in previous studies as presented in Table 3. [22]

**Table 6:** The energy conversion efficiency and physical characteristic of piezoelectric transducer type QB220-503YB chosen for this study [23].

PN	PM	W	S	C	RTP	RF	OCV	CCC	ROP
		grams	N/m	nF	(mm_peak_)	(Hz)	(Vpeak)	(μApeak / Hz)	(mW_rms_)
**Q220-A4-503YB**	5A4E	9.5	2.4x10^2^	260	± 1.57	45	± 18.1	± 46	4.7

PN, Part Number; PM, Piezo Material; W, Weight; S, Stiffness; C, Capacitance; RTP, Rated Tip Deflection; RF, Rated Frequency; OCV, Open Circuit Voltage (At rated deflection, parallel operation); CCC, Closed Circuit Current (Per sinusoidal cycle, at rated deflection, parallel operation); ROP, Rated Output Power (At rated deflection and frequency).

It covers the range of sound level pressure (dB) recorded through this study as represented earlier in Table 4. From the data sheet of the selected transducer (QB220-503YB) sound power produced by one piezoelectric transducer by the following equation (2): Power = I * V (uW) where (I) is the electric current and (V) is the voltage of the piezoelectric transducer [22]. Then it is converted to electric power using the equation (3): Converted Electric power = 10 log *P* (dBuW) where (*P*) is the power calculated in equation (2). dBW or decibel-watt is a unit of power in decibel scale, referenced to 1 watt (W) and uW is equal to 10^−6^ watt, thus in equation (4) will convert (dBuW) to (uW) by P(uW) = 1W ⋅ 10^(P(dBuW)/ 10)^ [35].

Electric energy is then calculated by the equation (5) for one transducer: Δ Electric Energy (one transducer) = (Electric power calculated in step (4) * 10–6 * Average time) (watt sec) or (Joules) [35], where the average time (2 hours) is the time of taking the measurements of noise levels of total 12 hours in 6 time interval. Then to calculate the total converted energy produced from known number of transducers can be calculated through the following equation (6): Total Electric Energy = No. of transducers X the electric energy produced by one transducer in equation (5).

Knowing that the LED street lamp consumes about 60 watt in an hour and about 780 watt through the 13 hours of evening and night intervals from (6 pm to 7 am) according to the Egyptian Environmental law 4/94. And the regular Halogen lamps consumes about 150 watt in an hour and about 1950 watt through the 13 hours of evening and night intervals. Calculating the number of street lamps that can be lit using the converted electric energy would be calculated by dividing the total amount of energy produced to the amount of energy consumed by one street lamp of any type.

## Results

**Fig 8:**
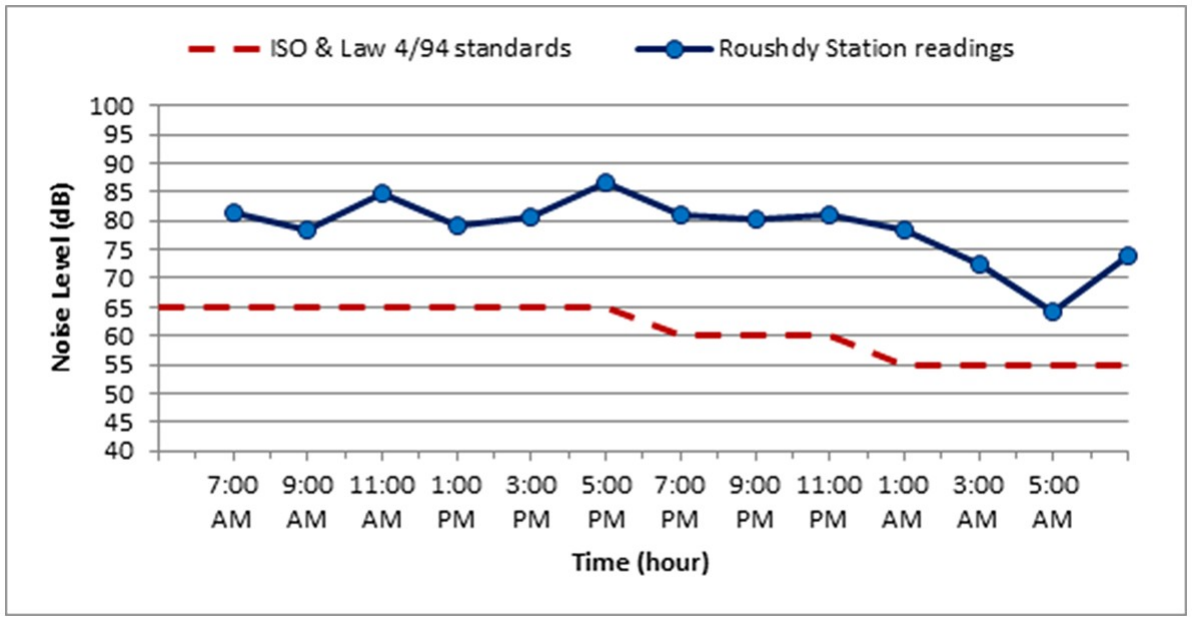
The variation of noise levels at ROS along the different intervals of the day compared to the standard permissible limit for noise levels according to ISO standards and Egyptian Environmental law 4/94.

**Fig 9:**
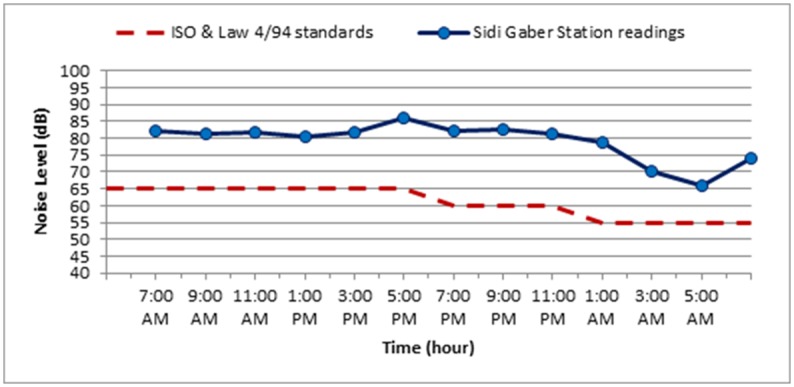
The variation of noise levels at Sidi Gaber Station along the different intervals of the day compared to the standard permissible limit for noise levels according to ISO standards and Egyptian Environmental law 4/94.

**Fig 10:**
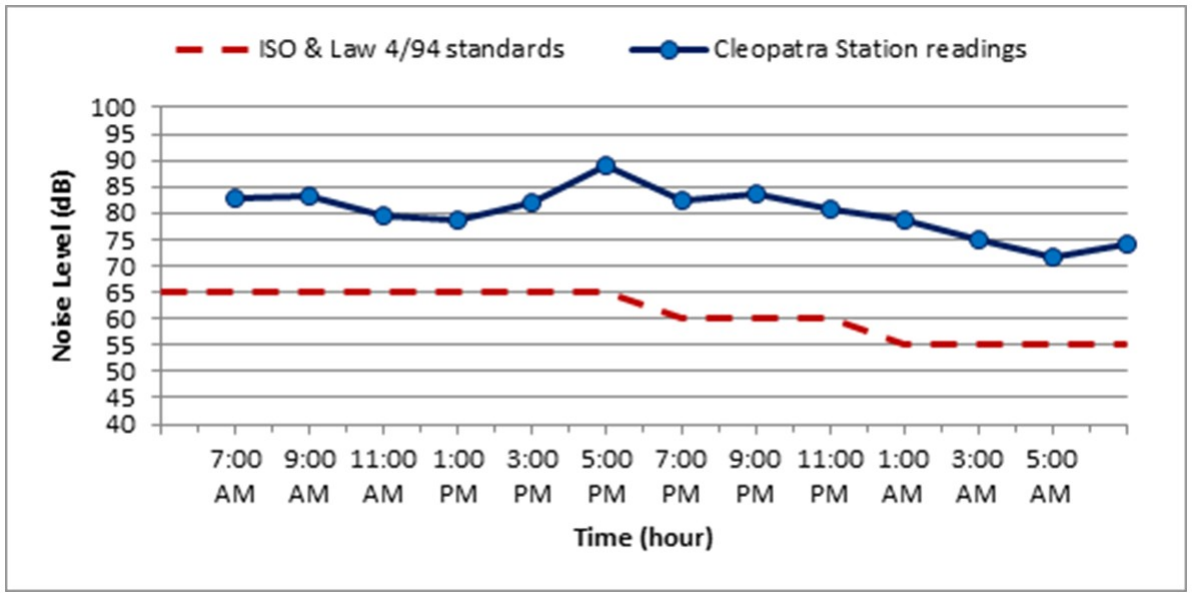
The variation of noise levels at Cleopatra Station along the different intervals of the day compared to the standard permissible limit for noise levels according to ISO standards and Egyptian Environmental law 4/94.

**Fig 11:**
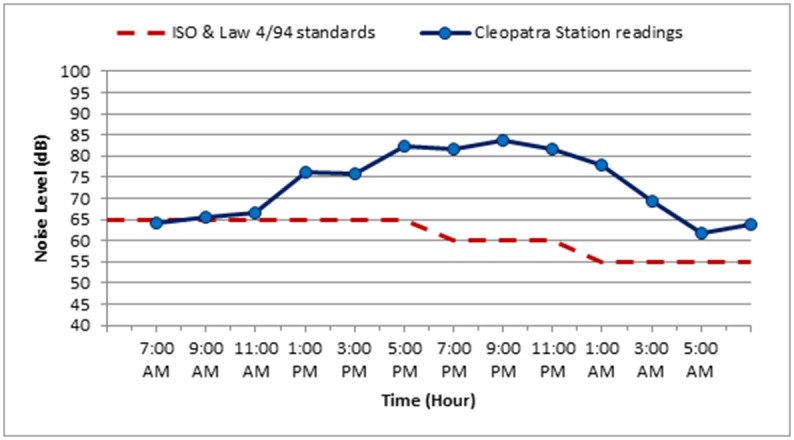
The variation of noise levels at Cleopatra Station on Weekend along the different intervals of the day compared to the standard permissible limit for noise levels according to ISO standards and Egyptian Environmental law 4/94.

The noise level measurements were carried out during October 2018 for the three selected stations along Elgeish Street in Alexandria City, Egypt. A typical data sheet was created for the selected stations as presented in Tables 7–10 in addition to Figs 8–11. The noise level measuring was taken in six separated intervals of the day, each interval is two hours and the noise level was recorded each 15 minutes in these 2 hours. The longitude and latitude position of each selected location were determined and mentioned in the table corresponded to each station.

**Table 7:** The data sheet for Roushdy Station (ROS) showing the noise level readings that were taken at the station on the selected intervals of the working day.

Station	Timing	Intervals	Readings (every 15 mins. interval)								Notes
**ROS**	Day	D1	81.4	79.2	85.1	83.2	79.5	83.2	83.3	78.5	Number of Lanes are 12. Coordinates: (31ʹ 23ʺ 30° N, 29ʹ 94ʺ 61° E).
		D2	84.9	81.3	84.5	83.7	80.1	81.5	81.7	79.2	
		D3	80.7	81.5	83.6	88.3	89.3	86.5	86.3	86.6	
	Evening	E1	80.9	81.3	81.1	84.4	83.7	78.3	81.1	80.2	
	Night	N1	81.2	80.3	81.1	81.3	80.7	79.8	78.7	78.6	
		N2	72.3	70.1	70.3	68.9	67.2	66.3	65.7	64.2	

**Table 8:** The data sheet for Sidi Gaber Station (SGS) showing the noise level readings that were taken at the station on the selected intervals of the working day.

Station	Timing	Intervals	Readings (every 15 mins. interval)								Notes
**SGS**	Day	D1	82.3	78.7	86.3	77.3	82.6	81.7	83.6	81.2	Number of Lanes are 10. Coordinates: (31ʹ 22ʺ 86° N, 29ʹ 94ʺ 17° E).
		D2	81.7	81.1	82.7	82.3	79.7	79.2	80.1	80.3	
		D3	80.3	81.7	83.5	87.3	88.4	85.6	85.7	85.9	
	Evening	E1	82.3	81.9	83.2	81.3	84.5	82.7	82.5	82.7	
	Night	N1	81.2	80.7	81.3	79.8	80.1	80.7	79.8	78.7	
		N2	70.4	70.3	68.3	67.9	68.3	66.9	65.3	65.9	

**Table 9:** The data sheet for Cleopatra Station (CLS) showing the noise level readings that were taken at the station on the selected intervals of the working day.

Station	Timing	Intervals	Readings (every 15 mins. interval)								Notes
**CLS**	Day	D1	82.7	79.4	85.7	82.7	77.2	86.7	85.9	83.3	Number of Lanes are 9. Coordinates: (31ʹ 22ʺ 51° N, 29ʹ 93ʺ 56° E).
		D2	79.6	77.9	78.1	79.3	78.4	77.8	80.4	78.7	
		D3	81.8	82.6	83.7	89.5	89.7	88.6	88.3	89.2	
	Evening	E1	82.6	81.2	83.6	82.9	82.0	83.2	84.4	83.8	
	Night	N1	80.6	82.3	81.7	80.5	80.3	81.9	79.9	78.7	
		N2	74.8	74.3	73.2	74.9	72.2	72.9	71.5	71.7	

**Table 10:** The data sheet for Cleopatra Station (CLS) showing the noise level readings that were taken at the station on the selected intervals during weekend.

Station	Timing	Intervals	Readings (every 15 mins. interval)								Notes
**CSL Weekend**	Day	D1	64.2	63.9	65.2	64.2	65.7	64.7	66.9	65.9	Number of Lanes are 9. Coordinates: (31ʹ 22ʺ 51° N, 29ʹ 93ʺ 56° E).
		D2	66.7	65.9	70.2	73.6	72.7	75.2	75.7	76.2	
		D3	75.8	74.2	76.7	78.3	81.1	80.7	83.7	82.2	
	Evening	E1	81.6	82.4	82.6	81.8	82.4	83.6	83.4	83.8	
	Night	N1	81.8	80.6	79.9	80.2	78.5	77.3	77.0	77.8	
		N2	69.3	65.3	61.1	62.2	61.3	60.4	60.8	61.9	

The mean equivalent, maximum and minimum noise levels at each of the selected location were calculated and represented in Table 11 for ROS, SGS and CLS on working days, and CLS on weekend.

**Table 11:** The mean equivalent sound pressure level (L_eq_), the maximum sound pressure level (Lmax) and the minimum sound pressure level (L_min_) in (dB) at each of the selected stations.

Station	Day timing	L_eq_ (dB)	L_max_ (dB)	L_min_ (dB)
**ROS**	Day	84.06	97.7	75.2
	Evening	81.75	92.5	76.1
	Night	77.60	88.9	61.3
**SGS**	Day	83.41	96.8	75.6
	Evening	82.73	92.8	78.8
	Night	77.61	87.8	60.1
**CLS**	Day	84.77	95.2	75.2
	Evening	83.07	95.0	77.1
	Night	78.58	89.3	68.9
**CLS Weekend**	Day	76.30	86.4	52.3
	Evening	82.67	91.3	70.6
	Night	76.55	88.3	51.2

The maximum noise level value at ROS was 97.7 dB at time interval 3–5 pm while the minimum noise level value was 61.3 dB at time interval of 3–5 am. In SGS the maximum level value was 96.8 dB at the interval of 3–5 pm and the minimum level value was 60.1 dB at interval of 3–5 am. And at CLS the maximum noise level value was 95.2 dB at time interval of 3–5 pm and the minimum noise level value was 68.9 dB at time interval of 3–5 am on the working day while on Friday the weekend the maximum noise level value was 91.3 dB at time interval 7–9 pm and the minimum noise level value was at time interval of 3–5 am with 51.2 dB.

It is noticed that the maximum noise level at the three stations was in the time interval of 3–5 pm on the working days as it is the rush hour of traffic and the maximum noise level is higher at CLS as it was monitored on Thursday the last working day before weekend with high traffic density and the width of the Street at CLS is smaller than the width of the Street at the other two stations. But on Friday the weekend at CLS, it is noticed that the maximum noise level was at the interval 7–9 pm as there is no schools or work that causes the high traffic on working days and most of citizens’ activates start at evening time.

The day-evening-night noise level index L_DEN_ was calculated for each selected station. The mean equivalent, maximum and minimum value of L_DEN_ noise levels were calculated by using equation (1) and presented in Table 12.

**Table 12:** The mean equivalent value of LDEN noise levels at each of the three selected stations.

Station	Index	Equivalent (dB)
ROS	L_DEN_	74.78
SGS	L_DEN_	74.96
CLS (On working day)	L_DEN_	75.79
CLS (On weekend)	L_DEN_	74.54

The L_DEN_ for the permissible limit of noise was calculated using equation (1) ${\text{L}}_{\text{DEN}}=10\;\text{log}\frac{1}{24}{\left(11*10^{L_{D}}+4*10^{L_{E+5}}+9*10^{L_{N+10}}\right)}^{\frac{1}{10}}$ (dB) [34]to be used to compare the L_DEN_ of the three selected stations that was calculated and presented in Table 11 and the resulted permissible equivalent L_DEN_ is 52.60 dB and presented in Table 13.

**Table 13:** The comparison between the permissible limit for noise levels allowed by the International Organization for Standardization (ISO) [30] [31] [32] and the Egyptian Environmental Law No. 4/1994 [13] and the equivalent mean LDEN calculated by equation (1) in Table 12.

Day timing	PL noise (dB)	Calculated L_DEN_ (dB)	Equivalent L_DEN_ at ROS (dB)	Equivalent L_DEN_ at SGS (dB)	Equivalent L_DEN_ at CLS (dB) (Working day)	Equivalent L_DEN_ at CLS (dB) (Weekend)
Day	65	52.60	74.78	74.96	75.79	74.54
Evening	60					
Night	55					

The results show a rise in the noise levels at the three stations through the whole day timing than the permissible noise limits. At ROS the equivalent noise level is higher than the equivalent permissible noise level **L**_**DEN**_ (52.6 dB) with 22.18 dB and in SGS is higher by 22.36 dB also in CLS is higher with 23.19 dB on working day and with 21.94 dB on the weekend.

**Fig 12:**
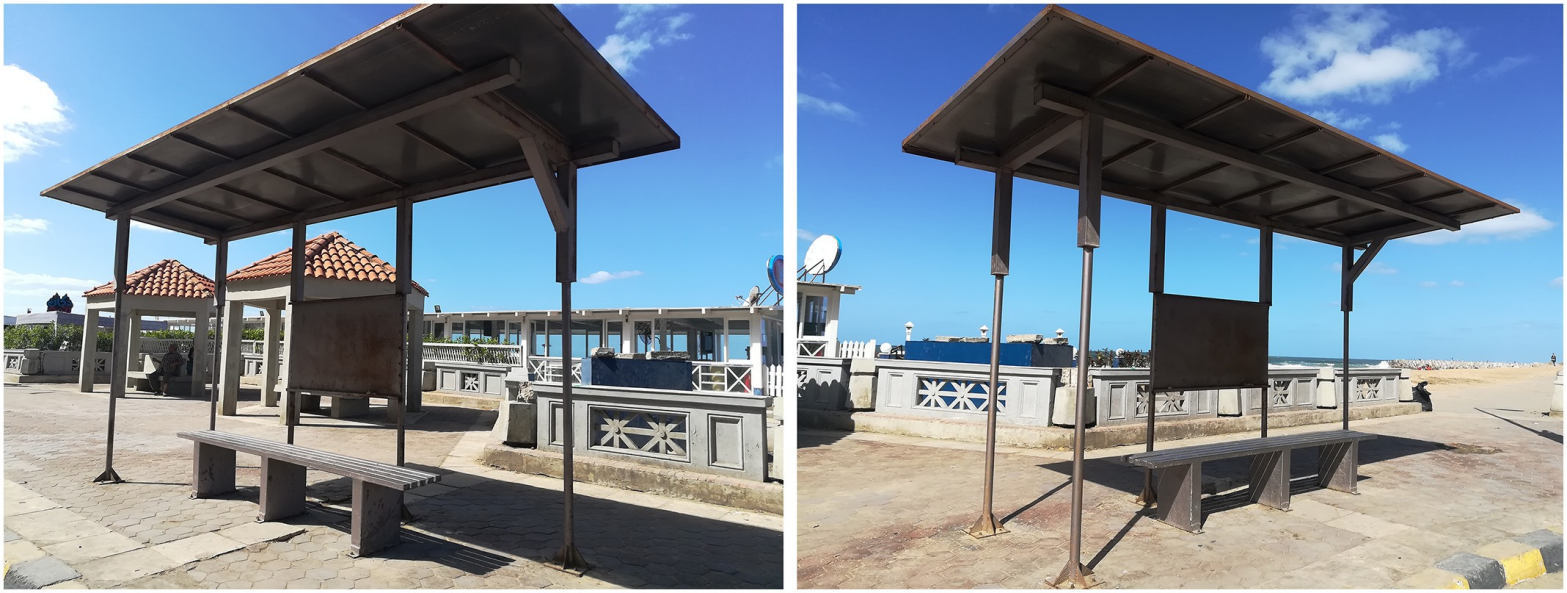
The bus stop at Cleopatra Station poor conditions as an illustration for other bus stops’ conditions. (Researcher, 2018).

**Fig 13:**
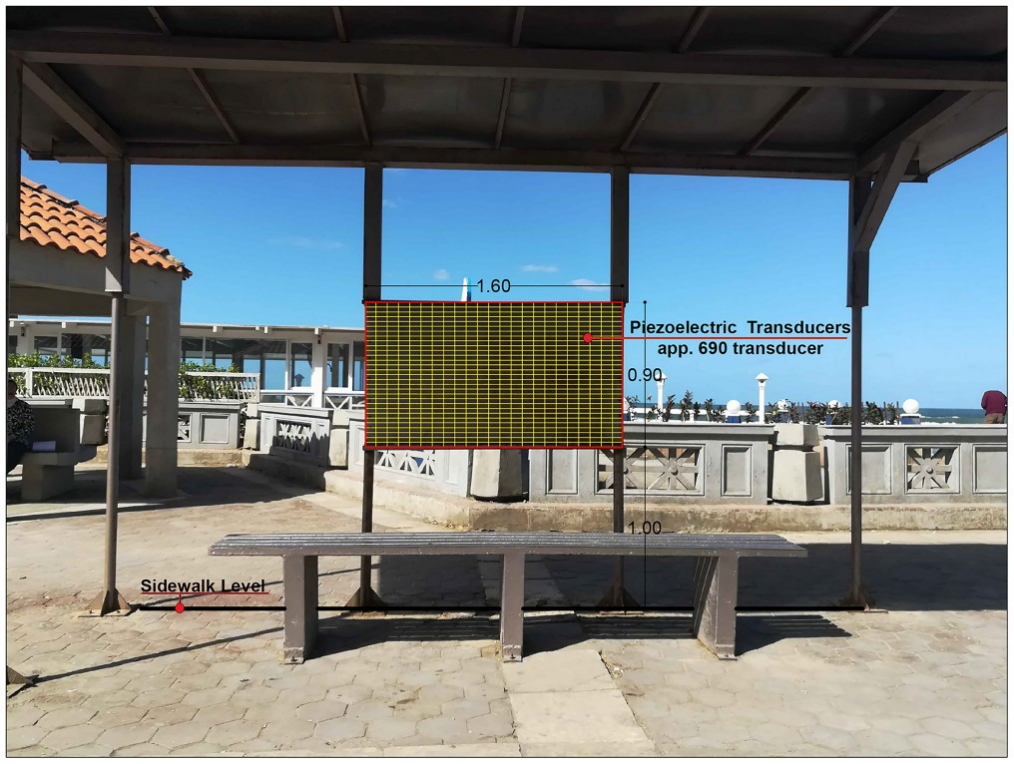
The proposed area to place the piezoelectric transducers at the bus stop of Cleopatra Station as an example. The area is about 1.45 m^2^ and it takes about 690 QB220-503YB transducers to cover this selected area (Researcher, 2018).

Applying the piezoelectric material transducer QB220-503YB with dimensions (31.8*66.7*1.8 mm) shown in Fig 7 at each of the three selected stations, utilizes the noise and convert it in to beneficial electric energy and Table 4 which was mentioned before represents the data sheet for the selected transducer properties. At each of the selected station the readings were taken at the bus stop. The bus stop stations have very poor and deteriorated structure as shown in Fig 12. The study proposes a renovation for the bus stop by applying the piezoelectric material transducers at the presented place in Fig 13 to convert the noise pollution in to electric energy and to develop the bus stop.

After applying the piezoelectric transducer QB220-503YB at the proposed area at each of the selected three stations and calculating the converted electric energy using the equivalent L_DEN_ at each station as a proposed trial to find if this conversion process is beneficial or not is presented in Tables 14 and 15 using the equations 2, 3, 4, and 5. As the three stations have equivalent L_DEN_ more than 80 dB, the data of the material when exposed to 80 dB will be considered and used in the calculations.

**Table 14:** The conversion process of noise to electric energy produced by one piezoelectric transducer of QB220-503YB at the selected stations through the data sheet presented in Table 4 and equations 2, 3, 4 and 5.

Equations	Stations			
	ROS	SGS	CLS (Working day)	CLS (Weekend)
Equivalent L_DEN_ (dB)	74.78	74.96	75.79	74.54
Equation 2: Power = I * V (uW)	0.9713 (uW)	0.9713 (uW)	0.9713 (uW)	0.9713 (uW)
Equation 3: Converted Electric power = 10 log *P* (dB uW)	-0.1265 (dB uW)	-0.1265 (dB uW)	-0.1265 (dB uW)	-0.1265 (dB uW)
Equation 4: Converting (dB uW) to (uW) P(uW) = 1W ⋅ 10^(P (dB uW)/ 10)^	0.97 uW	0.97 uW	0.97 uW	0.97 uW
Equation 5: Δ Electric Energy (of one transducer) = (Electric power calculated in step (4) * 10^−6^ * Average time (2 hours)) (W sec) or (Joules)	0.07 (W sec)	0.07 (W sec)	0.07 (W sec)	0.07 (W sec)

**Table 15:** The Total Electric energy produces by 690 transducers that are used to cover the proposed area at the bus stop at each of the three selected stations presented earlier in Fig 14 and using equation (6).

Equations	Stations			
	ROS	SGS	CLS (Working day)	CLS (Weekend)
Equation 6: Total Electric energy = No. of transducers (690) X Electric energy produced of one transducer in eq.(5) = (0.007)) (W sec) or (Joules)	4.83 W sec	4.83 W sec	4.83 W sec	4.83 W sec

From the observation to the stations recorded data sheet, it is noticed that the noise levels are higher than 70 dB at most of the day intervals except the hours from (1 am- 7 am) through night timing interval, thus the 690 transducers at the 3 stations will produce about 87-watt sec. in 18 hours (7 am- 1 am), subsequently 0.024-watt hour. As known a LED street lamp consume about 60 watts in one hour; so the produced amount of converted energy is not sufficient to run any application. Thus, the area used for the application needed to be increase to with about 2483 transducer units where each unit consists of 690 transducers to light 1 LED street lamp for only one hour.

## Discussion

On measuring and assessing the noise levels at three stations (ROS, SGS and CLS) along Elgeish road which is one of the major axis in Alexandria City, Egypt, using digital sound meter device and the selected measuring intervals according to International Organization for Standardization (ISO) [30] [31] [32] and the Egyptian Environmental Law No. 4/1994 [13].

The results show that the noise levels in all of the three selected stations exceeded the permissible limit for noise levels allowed by the Environmental Egyptian Law No. 4/1994. At the three stations the maximum noise level values were recorded at time interval of 3–5 pm and the highest value was at ROS with 97.7 dB on a working day, while the minimum noise level values were recorded at time interval of 3–5 am and the lowest value was at SGS with 60.1 dB on a working day. As for the weekend CLS was monitored as a sample to compare between noise levels on working days and weekend. Generally the noise levels through weekend is lower than working days, only the evening time interval (7–9 pm) could be nearly the same with working day noise levels.

In comparing the calculated results of SGS as an example to the results calculated by Zekry F. Ghatas in 2008 through his study of the assessment and analysis of traffic noise pollution in Alexandria city, Egypt; the maximum noise level value was 90.4 dB, the minimum noise level value was 60.2 dB and the mean equivalent noise level was 80.3 dB while in this study they were 96.8 dB, 60.1 dB and 86.67 correspondingly. It is notices that there is a remarkable increase in the noise levels through the past 10 years and this could be subjected to the massive change in the land use and layout of Elgiesh road that occurred in this station.

Thus, utilizing the noise pollution using the piezoelectric material concluded to be an applicable experiment. The electric energy produced from the proposed place of area 1.45 m2 containing 690 piezoelectric QB220-503YB transducers at the selected stations with LDEN higher than 70 dB was about 0.024 watts in an hour. And to make this amount of energy beneficial the area of the application needs to be maximized to at least with 2483 transducer units which need very large application area to make the amount of produced energy usable in lighting 1 LED street lamp for 1 hour, or it can be used in powering street advertisements and small devices or it can be stored and used when needed in applications that use greater amount of electric energy.

And referencing to Piezo Systems Company the unit price for one transducer of QB220-503YB is 265$, for 5 transducers is 186$, for 25 transducers is 144$, for 100 transducers is 127$, meaning that the total cost of 2483 transducer units (where each unit consists of 690 transducers costs 889$ as they considered approximately 7 groups of 100 transducers which cost 127$) at each station is about 2,207,390$ [36] and this proposal cost considered to be very expensive compared to the small amount of energy produced by these number of transducers and short powering interval, in addition to that it would take a long time to give its payback.

**Fig 14:**
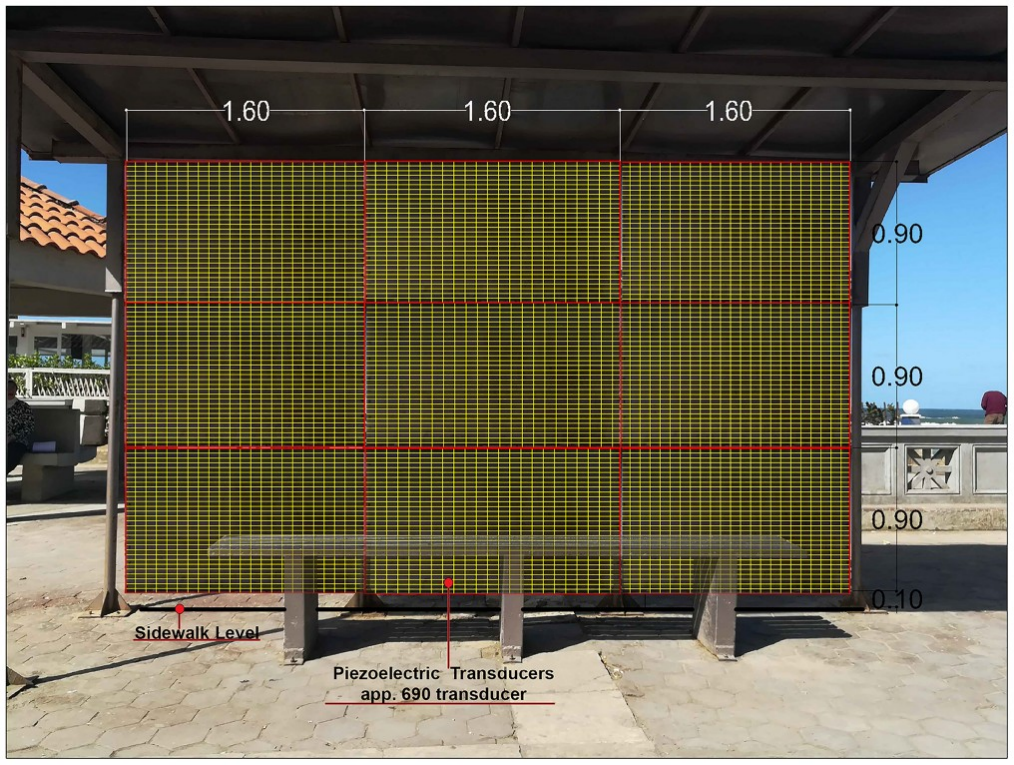
The second proposed area to place the piezoelectric transducers at the bus stop of Cleopatra Station as an example. The area is about 13 m2 and it takes about 6210 QB220-503YB transducers to cover this selected area (Researcher, 2018).

As a second proposal, the total area of applying the piezoelectric transducers at each station is maximized to the largest available area at the station which is 9 times the area used in the first proposal as presented in Fig 14. The total area in this proposal is 13 m2 containing 6210 piezoelectric QB220-503YB transducers producing about 0.22 watt in one hour and to make this amount of energy beneficial the area of the application at this proposal needs to be maximized to at least with 273 transducers units to produce energy that can be used in lighting 1 LED street lamp for 1 hour. The total cost of the 273 transducer units at each station is about 2,184,273$ which is also very high cost compared to the amount of the energy produced from them.

But comparing between the two proposals’ areas of applying the piezoelectric transducers, it is found that as the applying area increase the amount of converted produced energy increased and the total cost decreased.

Therefore, it is recommended to do more studies on applying the technology of piezoelectric materials with different piezoelectric transducers or by trying other aligning ways with large applying areas to utilize noise on vast application areas and in different locations with high traffic density such as city centers, city nodes and other main traffic roads; as noise considered a fast growing environmental problem and creates a great source of clean energy as well. In addition to increasing the production of the piezoelectric material to lower its cost as it form a great approach of producing clean energy.
